# Art-based interventions and non-motor outcomes in Parkinson's disease: a systematic review and meta-analysis

**DOI:** 10.3389/fpsyg.2026.1764300

**Published:** 2026-06-05

**Authors:** Zexi Liu, Silin An, Shuhan Liu, Qing Wang, Bin Jiang

**Affiliations:** 1 School of Media and Communications, Anyang Normal University, Anyang, China; 2 College of Arts, Jeonbuk National University, Jeonju-si, Republic of Korea; 3 Yanjing Institute of Technology, Langfang, China; 4 Nanhai College of Art and Technology, Haikou University of Economics, Haikou, China

**Keywords:** art-based interventions, fear of falling, non-motor symptoms, Parkinson's disease, rehabilitation

## Abstract

**Objective:**

This meta-analysis aimed to evaluate the effects of art-based interventions on non-motor symptoms among individuals with Parkinson's disease.

**Method:**

RCTs published between 1990 and September 30, 2025, were identified through searches in PubMed, Embase, Web of Science, and the Cochrane Library. Data extraction and quality assessment followed PRISMA guidelines using the Cochrane RoB 2.0 tool. Meta-analyses were conducted in R, with primary outcomes synthesized using forest and funnel plots, subgroup analyses, leave-one-out analysis and prediction intervals.

**Results:**

A total of 22 studies involving 928 participants were included. Art-based interventions produced a significant improvement in fear of falling and activity-related confidence (FES: *SMD* = −0.41; 95% CI [−0.67, −0.15]; *p* < 0.05; *I^2^* = 3.8%), with subgroup analyses showing a moderate effect for music interventions. The prediction interval excluded the null (95% PI [−0.78, −0.04]), indicating that the effect was relatively consistent across studies. Art-based interventions showed a small, non-significant effect on quality of life (PDQ-39: *SMD* = −0.19, 95% CI [−0.39, 0.01], *p* > 0.05). Subgroup analyses indicated that combined music and dance–music interventions yielded the strongest point estimates and reached statistical significance, although these findings were derived from single studies. Theater and dance interventions did not reach statistical significance for quality-of-life outcomes. No significant improvements were observed in cognitive function (MoCA: *SMD* = 0.05; 95% CI [−0.19, 0.30]; *p* > 0.05), and depressive symptoms showed only a small, non-significant reduction (BDI: *SMD* = −0.23; 95% CI [−0.63, 0.18]; *p* > 0.05).

**Conclusion:**

Art-based interventions showed selective benefits for non-motor symptoms in Parkinson's disease, with significant reductions in fear of falling, especially in music-based approaches. Effects on quality of life were small and non-significant, and no clear improvements were found for cognition or depression. Larger trials are needed to confirm clinical relevance.

**Systematic review registration:**

Identifier: CRD42025633292.

## 1 Introduction

Parkinson's disease (PD) is a prevalent neurodegenerative disorder (Gourie-Devi, 2014) predominantly affecting older adults, though it can also occur in younger populations. It is characterized by postural instability and motor impairments (Lord et al., 2013), which significantly compromise the quality of life of those affected (Bloem et al., 2001). Motor symptoms of PD include resting tremor, rigidity, bradykinesia, akinesia, gait disturbances, postural instability, and respiratory dysfunction. These symptoms collectively contribute to a heightened risk of falls and a significant decline in functional independence. Non-motor symptoms of PD affect almost all bodily systems, including gastroparesis, constipation, anxiety, depression, fatigue, REM sleep behavior disorder, hyposmia or anosmia, dysarthria (Bloem et al., 2021; Chaudhuri et al., 2006), and a range of mood, cognitive, psychiatric, and behavioral abnormalities (D'Iorio et al., 2021; Maggi et al., 2023; Sapir et al., 2011). These manifestations are associated with social withdrawal and a reduction in quality of life (Chaudhuri et al., 2006; Martinez-Martin et al., 2015).

The conservative management of PD primarily relies on pharmacological treatments that alleviate symptoms. However, these treatments do not fully address all clinical signs, particularly non-motor symptoms (Pantcheva et al., 2015). Additionally, side effects and motor complications often limit the effectiveness of these treatments, both in terms of dosage and duration. As the disease advances, patients experience more severe symptoms, and medications become less effective in managing these issues, greatly increasing the difficulty of managing advanced stages (Ishihara et al., 2007; Xu et al., 2014). Therefore, alternative treatments from specialists in various fields are becoming increasingly important in improving both symptoms and patients' quality of life. Among non-pharmacological treatments, physiotherapy is the most widely used.

Research has shown that physiotherapy can significantly improve motor symptoms, gait, and balance in Parkinson's patients, showing greater evidence-based efficacy than many other non-pharmacological interventions (Liu et al., 2023; Radder et al., 2020). However, physiotherapy does not address non-motor symptoms, which are often neglected but have a profound impact on emotional and cognitive health, making them a crucial area for treatment.

These limitations have prompted increased attention to the non-motor symptoms of PD. In addition, the high cost, diminishing long-term efficacy, and considerable side-effect burden associated with pharmacological treatment have intensified the need for safe, accessible, and cost-effective adjunctive approaches. Evidence from other clinical populations indicates that art-based interventions can deliver meaningful psychosocial and functional benefits (Bloem et al., 2004; Pringsheim et al., 2014; Liu et al., 2025), raising the question of whether similar advantages may be achievable for individuals with PD. Against this background, art-based interventions have gained growing interest as modalities capable of targeting domains that conventional rehabilitation seldom improves, including mood, cognition, quality of life, and fall-related self-efficacy. However, the current evidence remains fragmented and lacks systematic integration. Existing meta-analyses have primarily focused on motor outcomes or have been restricted to specific modalities such as dance or active group-based performing arts, with no comprehensive quantitative synthesis specifically addressing non-motor symptoms in Parkinson's disease.

These limitations in the current evidence base underscore the theoretical importance and clinical relevance of synthesizing current evidence on art-based interventions for non-motor symptoms in PD. Therefore, the present study undertakes a systematic review and meta-analysis to evaluate their therapeutic effectiveness across major non-motor domains.

In this review, Art-based interventions refer to structured, non-pharmacological activities grounded in artistic modalities (e.g., music, dance, drama, and visual arts) delivered with therapeutic intent, consistent with the WHO arts and health framework (World Health Organization, 2019). This construct is broader than Art-Based Interventions, which AATA and EFAT describe as a mental health profession practiced within a psychotherapeutic relationship (European Federation of Art Therapy, n.d.; American Art Therapy Association, n.d.; Canning et al., 2015). Mind–body interventions (e.g., yoga and mindfulness) were identified but analyzed separately to avoid conceptual overlap.

## 2 Method

This meta-analysis was conducted in accordance with the Cochrane Handbook for Systematic Reviews of Interventions, and findings were reported following the Preferred Reporting Items for Systematic Reviews and Meta-Analyses (PRISMA) guidelines. The protocol was prospectively registered in PROSPERO (CRD42025633292). All statistical analyses were performed in R (version 4.1.2, meta package), including random-effects meta-analyses with forest and funnel plots, leave-one-out sensitivity analyses, prespecified subgroup analyses, and calculation of 95% prediction intervals to assess robustness and between-study heterogeneity.

### 2.1 Search strategy

A thorough literature search was performed across multiple electronic databases, including the Cochrane Library, Web of Science, PubMed, and EMBASE, covering publications from their inception up to September 30, 2025. Using a comprehensive search strategy that combined Medical Subject Headings (MeSH) and free-text terms, we screened titles, abstracts, keywords, and subsequently conducted a full-text review. The temporal range of this review spanned from January 1990 to September 30, 2025, with non-English language literature excluded. These time and language restrictions were applied to focus the research team on the latest, relevant studies within their language proficiency, thereby enhancing the quality and validity of the findings. The search strategy, initially based on PubMed, was extended to additional databases. A manual search was also conducted by reviewing the bibliographies of pertinent articles and references within the selected studies (Table 1).

**Table 1:** Search strategy.

**Number**	**Search terms**
#1	Parkinson disease [MeSH]
#2	Idiopathic Parkinson's disease [Title/Abstract]
#3	Lewy body Parkinson's disease [Title/Abstract]
#4	Parkinson's disease idiopathic [Title/Abstract]
#5	Parkinson's disease lewy body [Title/Abstract]
#6	Parkinson disease idiopathic [Title/Abstract]
#7	Parkinson's disease [Title/Abstract]
#8	Idiopathic Parkinson disease [Title/Abstract]
#9	Lewy body Parkinson disease [Title/Abstract]
#10	Primary Parkinsonism [Title/Abstract]
#11	Parkinsonism primary [Title/Abstract]
#12	Paralysis agitans [Title/Abstract]
#13	#2 OR #3 OR #4 OR #5 OR #6 OR #7 OR #8 OR #9 OR #10 OR #11 OR #12
#14	#1 OR #2 OR #3 OR #4 OR #5 OR #6 OR #7 OR #8 OR #9 OR #10 OR #11 OR #12 OR #13
#15	Art-based intervention [MeSH]
#16	Art therapy” [Title/Abstract]
#17	Art Intervention” [Title/Abstract]
#18	Art Interventions” [Title/Abstract]
#19	Singing” [Title/Abstract]
#20	Dancing” [Title/Abstract]
#21	Painting”[Title/Abstract]
#22	Drawing” [Title/Abstract]
#23	Calligraphy” [Title/Abstract]
#24	Music” [Title/Abstract]
#25	Sing” [Title/Abstract]
#26	Paint” [Title/Abstract]
#27	Draw” [Title/Abstract]
#28	Dance” [Title/Abstract]
#29	Movement” [Title/Abstract]
#30	Dance movement” [Title/Abstract]
#31	Dance and movement” [Title/Abstract]
#32	Psychodrama” [Title/Abstract]
#33	Psycho - drama” [Title/Abstract]
#34	Psycho drama” [Title/Abstract]
#35	Sculpture” [Title/Abstract]
#36	Collage” [Title/Abstract]
#37	Poetry” [Title/Abstract]
#38	Drama” [Title/Abstract]
#39	Theater” [Title/Abstract]
#40	Theater” [Title/Abstract]
#41	Clay” [Title/Abstract]
#42	Visual arts therapy [Title/Abstract]
#43	Meditation therapy [Title/Abstract]
#44	Mindfulness [Title/Abstract]
#45	Yoga [Title/Abstract]
#46	Psychodrama [Title/Abstract]
#47	#16 OR #17 OR #18 OR #19 OR #20 OR #21 OR #22 OR #23 OR #24 OR #25 OR #26 OR #27 OR #28 OR #29 OR #30 OR #31 OR #32 OR #33 OR #34 OR #35 OR #36 OR #37 OR #38 OR #39 OR #40 OR #41 OR #41 OR #42 OR #43 OR #44 OR #45 OR #46
#48	#15 OR#16 OR #17 OR #18 OR #19 OR #20 OR #21 OR #22 OR #23 OR #24 OR #25 OR #26 OR #27 OR #28 OR #29 OR #30 OR #31 OR #32 OR #33 OR #34 OR #35 OR #36 OR #37 OR #38 OR #39 OR #40 OR #41 OR #41 OR #42 OR #43 OR #44 OR #45 OR #46
#49	Randomized controlled trial” [Publication Type]
#50	Trial” [Title/Abstract]
#51	Random^*^” [Title/Abstract]
#52	Randomized controlled trial” [Publication Type]
#53	#49 OR #50 OR #51 OR #52
#54	#14 AND #48 AND #53

MeSH, Medical Subject Headings.

### 2.2 Inclusion and exclusion criteria

#### 2.2.1 Inclusion criteria

The selection process followed the PICOS framework (Population, Intervention, Comparison, Outcome, and Study Design) to ensure methodological consistency and rigor.

**Population:** (1) Individuals clinically diagnosed with PD by qualified clinicians or established diagnostic guidelines. (2) Aged ≥ 40 years, capable of standing for at least 30 Seconds, and able to walk independently for a minimum of 3 meters (with or without assistive devices). (3) Participants with psychiatric disorders other than PD or those unable to continue observation due to severe physical illness were excluded.

**Intervention:** Any form of art-based intervention.

**Comparison:** Conventional rehabilitation, standard medical care, or other non-art-based interventions.

**Outcome:** Primary outcomes (art-based interventions) included quality of life (PDQ-39), fear of falling (FES), cognitive function (MoCA), and depressive symptoms (BDI). Secondary outcomes (yoga and mindfulness interventions) included quality of life (PDQ-39 and PDQ-8) and anxiety and depression (HADS).

**Study Design:** (1) Only randomized controlled trials (RCTs) published in English and reporting original clinical data. (2) Excludes case reports, editorials, reviews, conference abstracts.

#### 2.2.2 Exclusion criteria

(1) Studies without full-text available in English will be excluded.
(2) Non-randomized studies (e.g., observational studies, quasi-experimental designs), as well as reviews, study protocols, conference abstracts, case reports, animal studies, and online reports, will be excluded.
(3) Studies without sufficient quantitative data for effect size calculation will be excluded.
(4) Observational studies will be excluded.

Overall, these criteria ensure that only high-quality randomized controlled trials with sufficient data are included in the meta-analysis.

### 2.3 Study selection and data extraction

All records retrieved from database searches were imported into EndNote 21 for deduplication. Two independent reviewers screened titles and abstracts, followed by full-text assessments, to determine eligibility according to predefined inclusion and exclusion criteria. Discrepancies were resolved through discussion, and unresolved disagreements were adjudicated by a third reviewer. Inter-rater reliability for both screening stages was evaluated using Cohen's kappa coefficient and interpreted according to established guidelines.

Data extraction was independently conducted by the two reviewers using a standardized extraction form. Extracted variables included publication year, study location, diagnostic criteria, sample size, mean or median age, percentage of male participants, trial registration number, intervention characteristics, comparator details, and primary and secondary outcomes. When available, intention-to-treat data were prioritized over completer analyses. For studies with incomplete or unclear data, corresponding authors were contacted for clarification.

During data extraction, interventions were categorized based on definitions outlined in established guidelines (AATA). Art therapy modalities included drama, dance, music, and visual arts (painting), as well as multimodal interventions integrating multiple artistic components, such as dance-Qigong. This classification aligns with current WHO rehabilitation frameworks.

Only outcomes consistently and adequately reported across the included trials were eligible for quantitative synthesis. Although non-motor symptoms encompass several domains (e.g., sleep, fatigue, autonomic function, anxiety), most studies lacked extractable or standardized data for these measures. Consequently, meta-analysis was restricted to PDQ-39, FES, MoCA, and BDI, which were the only outcomes reported with sufficient statistical detail.

### 2.4 Quality assessment

The methodological quality of the included studies was evaluated using the Cochrane Risk of Bias 2.0 (RoB 2) tool. This instrument assesses five key domains:(1) the randomization process, (2) deviations from intended interventions, (3) missing outcome data, (4) measurement of outcomes, and (5) selection of the reported results.

Two independent reviewers conducted the risk-of-bias assessments, with discrepancies resolved through discussion. In cases where consensus could not be achieved, a third reviewer adjudicated the decision. Each study outcome was rated as having “low risk of bias,” “some concerns,” or “high risk of bias,” following the official RoB 2 guidelines.

**Figure 1:**
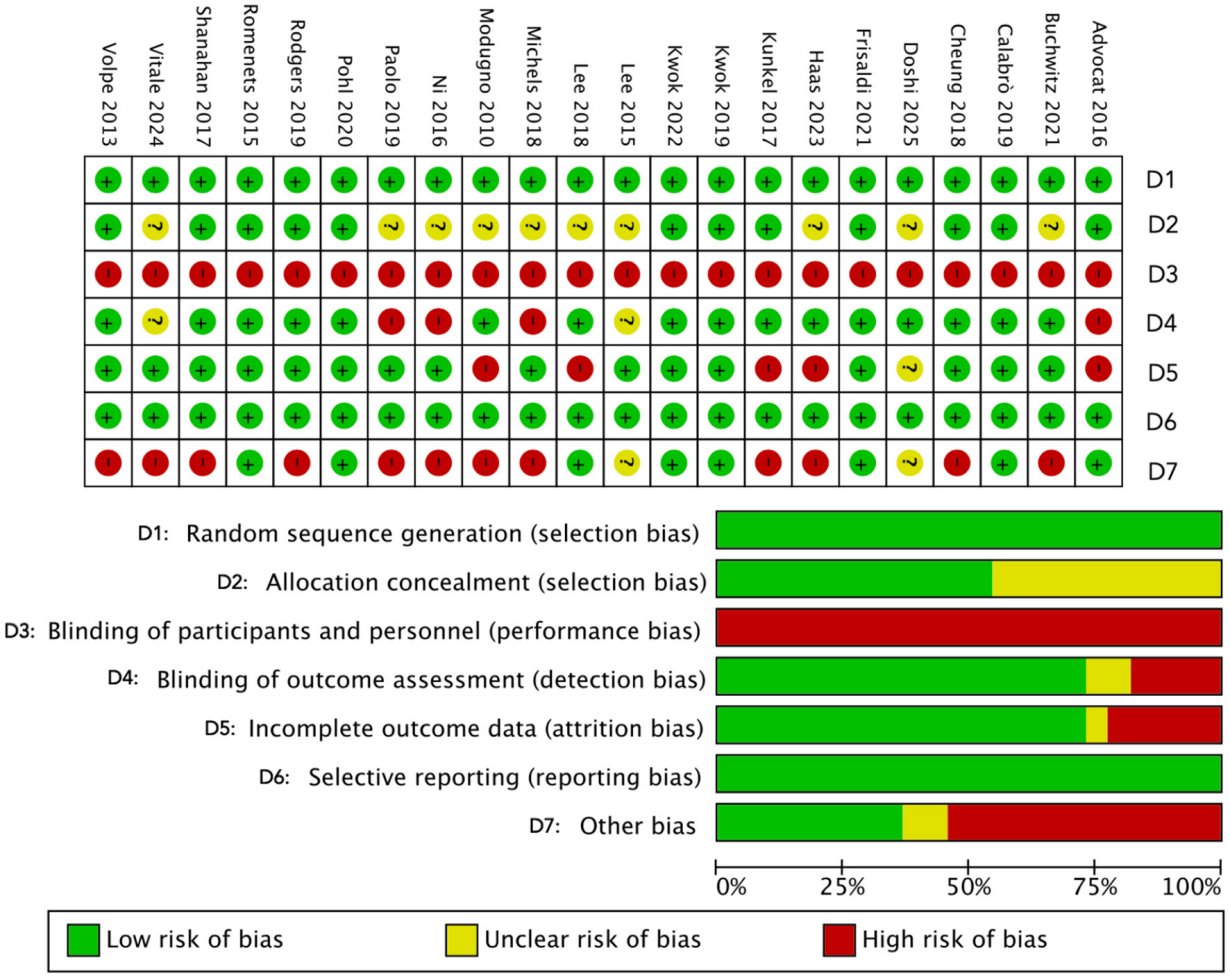
Risk of bias summary using the RoB-2 traffic-light visualization across included randomized controlled trials.

This systematic appraisal ensured a rigorous evaluation of internal validity and strengthened the overall reliability of the meta-analysis findings, particularly regarding the evidence base for non-motor outcomes in PD (Figure 1).

D1: For random sequence generation, 22 studies demonstrated a low risk of bias (Advocat et al., 2016; Buchwitz et al., 2021; Calabrò et al., 2019; Cheung et al., 2018; Doshi et al., 2025; Frisaldi et al., 2021; Haas et al., 2024; Kunkel et al., 2017; Kwok et al., 2022, 2019; Lee et al., 2018, 2015; Michels et al., 2018; Modugno et al., 2010; Ni et al., 2016; Paolo et al., 2019; Pohl et al., 2020; Rios Romenets et al., 2015; Rodgers et al., 2019; Shanahan et al., 2017; Vitale et al., 2024; Volpe et al., 2013), accounting for 100%.

D2: Concerning allocation concealment, 12 studies were found to have a low risk of bias (54.55%) (Advocat et al., 2016; Calabrò et al., 2019; Cheung et al., 2018; Frisaldi et al., 2021; Kunkel et al., 2017; Kwok et al., 2022, 2019; Pohl et al., 2020; Rios Romenets et al., 2015; Rodgers et al., 2019; Shanahan et al., 2017; Volpe et al., 2013), while the remaining 45.45% had an unclear risk.

D3: Twenty-two studies exhibited a high risk of bias in the blinding of participants and personnel, with no studies at low risk.

D4: For blinding of outcome assessment, 16 studies were assessed as having a low risk of bias (72.73%) (Buchwitz et al., 2021; Calabrò et al., 2019; Cheung et al., 2018; Doshi et al., 2025; Frisaldi et al., 2021; Haas et al., 2024; Kunkel et al., 2017; Kwok et al., 2022, 2019; Lee et al., 2018; Modugno et al., 2010; Pohl et al., 2020; Rios Romenets et al., 2015; Rodgers et al., 2019; Shanahan et al., 2017; Volpe et al., 2013), with 18.18% showing high risk and 9.09% remaining unclear.

D5: In terms of incomplete outcome data, 16 studies were found to have a low risk of bias (72.73%) (Buchwitz et al., 2021; Calabrò et al., 2019; Cheung et al., 2018; Frisaldi et al., 2021; Kwok et al., 2022, 2019; Lee et al., 2015; Michels et al., 2018; Ni et al., 2016; Paolo et al., 2019; Pohl et al., 2020; Rios Romenets et al., 2015; Rodgers et al., 2019; Shanahan et al., 2017; Vitale et al., 2024; Volpe et al., 2013), one study was rated as unclear risk (4.55%), and 22.72% were rated as high risk.

D6: Regarding selective reporting and outcome reporting, 22 studies showed a low risk of bias, making up 100%.

D7: For other types of bias, 8 studies exhibited a low risk (36.36%) (Advocat et al., 2016; Calabrò et al., 2019; Frisaldi et al., 2021; Kwok et al., 2022, 2019; Lee et al., 2018; Pohl et al., 2020; Rios Romenets et al., 2015), two studies were rated as unclear risk (9.09%), and 54.55% were found to have a high risk.

### 2.5 Assessment of evidence certainty using the GRADE approach

Although GRADE is recommended for evaluating the certainty of evidence, its application to arts-based randomized trials warrants careful interpretation. Trials in this field frequently involve relatively small sample sizes and limited feasibility of participant and provider blinding due to the nature of the interventions (Boutron et al., 2017), which may contribute to downgrading for imprecision and risk of bias under the GRADE framework. To maintain methodological transparency and consistency with contemporary evidence synthesis practice, GRADE assessments were conducted for all outcomes. These ratings should therefore be interpreted considering the methodological and practical characteristics typical of arts-based intervention research (Table 2).

**Table 2:** GRADE evidence profile.

**Outcome**	**k**	**N**	**SMD**	**95% CI**	**I^2^**	**Risk of bias (a)**	**Incon-sistency (b)**	**Indirect-ness (c)**	**Impreci-sion (d)**	**Publica-tion bias (e)**	**Certainty of evidence (GRADE)**	**Down-grading footnotes (a–e)**	**Reasons for downgrading**
**Art-based interventions**													NS: Not Serious; S:Serious
Quality of life (PDQ-39)	12	420	−0.19	[−0.39; −0.01]	18.40%	NS	NS	NS	S	S	Low●●○○	d, e	Confidence intervals crossed the threshold for clinical decision-making; Small total sample size
Fear of falling (FES)	4	242	−0.41	[−0.67; −0.15]	3.80%	NS	NS	NS	S	NS	Moderate●●●○	d	Small total sample size
Cognition (MoCA)	7	263	0.05	[−0.19; 0.30]	0%	NS	NS	NS	S	S	Low●●○○	d, e	Confidence intervals crossed the threshold for clinical decision-making; Small total sample size
Depression (BDI)	8	220	−0.23	[−0.63; 0.18]	51.90%	NS	S	NS	S	S	Very Low●○○○	b, d, e	Substantial heterogeneity; Confidence intervals crossed the threshold for clinical decision-making; Small total sample size
**Mind–body interventions**													NS: Not Serious; S: Serious
Quality of life (PDQ-39)	7	404	−0.38	[−0.72; −0.05]	54.40%	NS	S	NS	S	NS	Low●●○○	b, d	Substantial heterogeneity; Small total sample size
Quality of life (PDQ-8)	2	278	−0.64	[−0.88; −0.40]	0%	NS	NS	NS	S	NS	Moderate●●●○	d	Small total sample size
Anxiety (HADS)	2	278	−0.81	[−1.05; −0.56]	0%	NS	NS	NS	S	NS	Moderate●●●○	d	Small total sample size
Depression (HADS)	2	278	−0.91	[−1.16; −0.67]	0%	NS	NS	NS	S	NS	Moderate●●●○	D	Small total sample size

### 2.6 Data analysis

All statistical analyses were performed in R (version 4.1.2) using the meta package. Standardized mean differences and their standard errors were calculated for each study arm before pooling the results. Comparative effects between various Art-Based Interventions and their corresponding control groups were synthesized through pairwise meta-analyses. Between-study heterogeneity was evaluated using the Cochrane Q test and the I^2^ statistic, with values of 0-25%, 25-50%, and > 50% interpreted as low, moderate, and high heterogeneity. A random-effects model was applied when I^2^ exceeded 30%, whereas a common-effects model was used when heterogeneity was minimal. Statistical significance was defined as a two-sided *p* < 0.05. Publication bias was examined using Egger's regression test together with visual assessment of funnel plot asymmetry.

In addition to pooled effect estimates, 95% prediction intervals were computed to assess the potential range of true effects in future similar studies, offering a more conservative and clinically informative interpretation of between-study variability. Employing a 30% heterogeneity threshold allowed earlier identification of inconsistency across studies, enabling the use of random-effects modeling under moderate variability. Compared with the traditional 50% cutoff, this approach provides a more sensitive assessment of heterogeneity and enhances the robustness and interpretability of the meta-analytic findings. Effect sizes were interpreted according to Cohen's conventional thresholds small (*SMD* ≈ 0.2), medium (*SMD* ≈ 0.5), and large (*SMD* ≈ 0.8).

## 3 Results

### 3.1 Study identification

The database search initially identified 2,628 citations. After removing duplicates, 1,835 records remained for screening. Of these, 1,745 records were excluded after title and abstract screening due to reasons such as non-RCT design, literature reviews, meta-analyses, comments, unrelated topics, or incomplete publications. The remaining 90 full-text articles were assessed for eligibility. Among these, 22 were excluded due to non-relevant outcomes, 8 for non-compliant study design, 10 for incomplete or unsuitable full text, 6 for non-target populations, 13 for insufficient or inadequate data, and 9 for non-eligible interventions.

**Figure 2:**
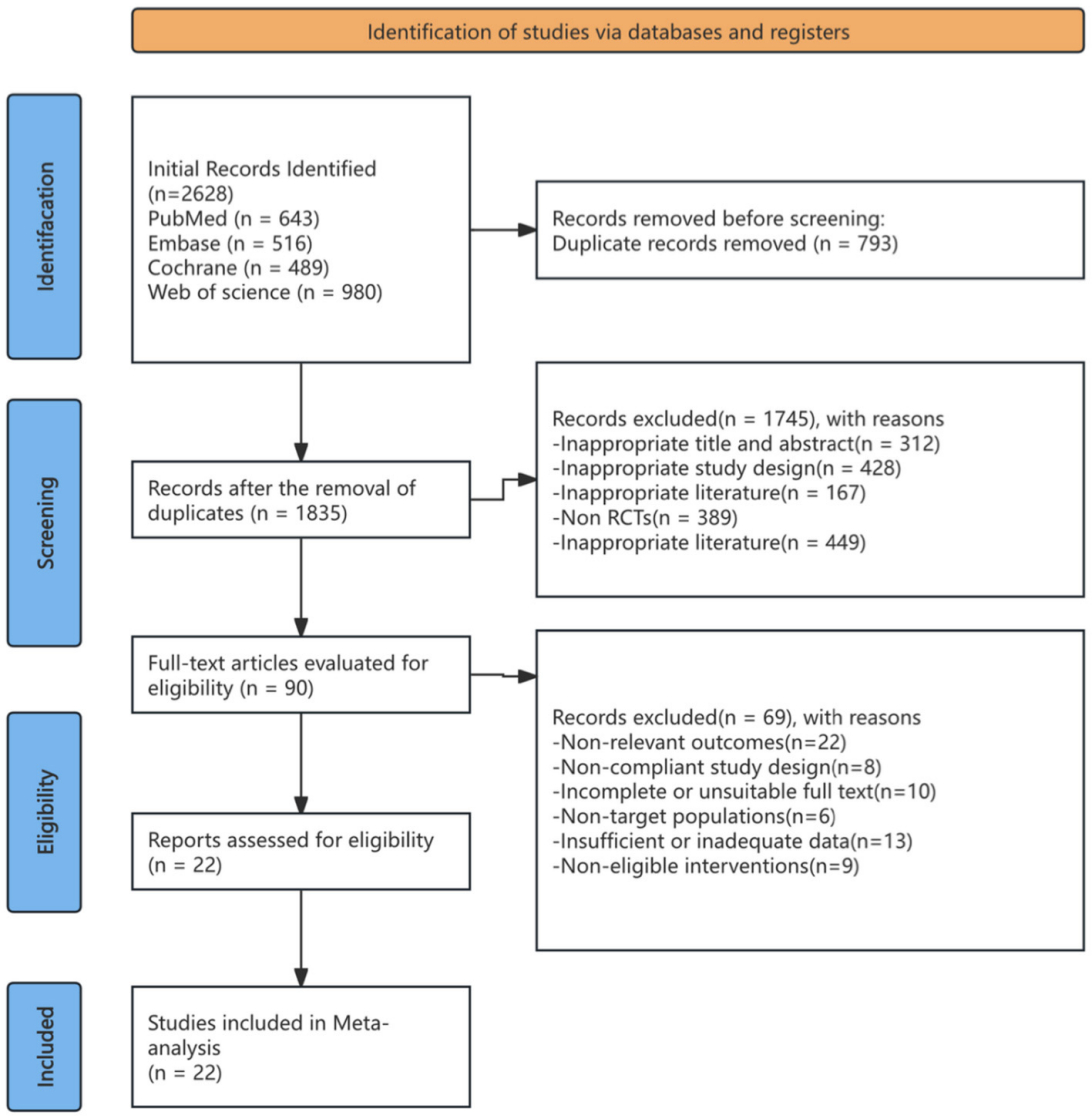
PRISMA flow diagram.

Ultimately, 22 studies met the inclusion criteria and were included in the systematic review and meta-analysis (Figure 2).

### 3.2 Study characteristic

This meta-analysis included 928 participants from 22 randomized controlled trials conducted between 1990 and September 30, 2025. All control groups no received Art-Based Interventions. The mean age of participants was 65.6 ± 5.3 years in the experimental groups and 66.9 ± 5.9 years in the control groups. The proportion of male participants was 54.94% in the experimental groups and 50.75% in the control groups (overall 55.68%). The mean duration of PD was 6.26 ± 3.90 years.

Among the included trials, 72% were registered with a trial registration number. Geographically, the studies were conducted in a range of countries: 6 in Italy, 3 in the United States, 1 in Sweden, 2 in China (including Hong Kong), 1 in Brazil, 1 in Ireland, 2 in Korea, 1 in Canada, 1 in India, 1 in the United Kingdom, 2 in Australia, and 1 in Germany.

### 3.3 Characteristics of the intervention

The types of art interventions varied: nine studies employed dance-based interventions, two implemented music-based interventions, and one applied a theater-based intervention. In addition, nine multimodal interventions were included. Training sessions ranged from 15 to 120 min, and overall intervention periods spanned 5 weeks to 3 years. Control interventions also varied: twelve studies applied passive control interventions (including four wait-list interventions and eight usual-care or no-intervention conditions), whereas eight studies implemented active control interventions, including physiotherapy interventions, home-based exercise interventions, treadmill walking (non-RAS) interventions, deep-water running interventions, Nordic walking interventions, stretching and flexibility interventions, resistance-training interventions, and multi-component exercise interventions. The PDQ-39 was applied in 12 studies, the BDI in 8 studies, the MoCA in 7 studies, and the FES in 5 studies, reflecting their frequent utilization for assessing quality of life, depressive symptoms, cognitive function, and fall efficacy, respectively (Table 3).

**Table 3:** Summary of included reviews.

**Study**	**Study design**	**Country**	**Types of art**	**Sample size**	**Gender (M)**	**Mean age**	**Disease duration**	**Funding registration**	**Intervention**		**Outcome**
				**EG, CG**	**EG, CG**	**EG, CG**	**EG, CG**		**Intervention content time, frequency, period(EG)**	**Intervention content time, frequency, period(CG)**	
Volpe et al. (2013)	RCT	Italy	Dance	EG:12 CG:12	58.33% 50%	61.6 ± 4.5 years 65 ± 5.3 years	9.0 ± 3.6 years 8.9 ± 2.5 years	2012-005769-11	Irish set dancing classes (1.5-h sessions once a week for 6 months)	Physiotherapy (1.5-h sessions once a week for 6 months)	PDQ39
Vitale et al. (2024)	RCT	Italy	Dance	EG:14 CG:14	85.71% 71.43%	64.1 ± 7.9 years 62.9 ± 4.7 years	5.5 ± 2.8 years 6.5 ± 3.5 years	N/A	Biodanza involves movement, music, and emotional experiences (2-h sessions once a week for 12 weeks)	No intervention or motor activity	PDQ39, BDI
Shanahan et al. (2017)	RCT	Ireland	Dance	EG:20 CG:21	65% 61.90%	69 ± 10 years 69 ± 8 years	5.5 ± 6 years 6 ± 8 years	NCT01939717	Irish set dancing (1.5-h sessions once a week for 10 weeks)	No additional intervention, maintaining routine care, and activities (Usual care and daily activities)	PDQ39
Rios Romenets et al. (2015)	RCT	Canada	Dance	EG:18 CG:15	66.67% 46.67%	63.2 ± 9.9 years 64.3 ± 8.1 years	5.5 ± 4.4 years 7.7 ± 4.6 years	NCT01573260	Argentine tango classes (Two 1-h sessions per week for 12 weeks)	Routine home exercises based on provided Parkinson's disease exercise guidelines (Daily self-guided exercise, no fixed time)	PDQ39, MoCA, BDI
Rodgers et al. (2019)	RCT	Australia	MBIs (Mindful-ness)	EG:15 CG:12	66.67% 61.11%	63.7 ± 8.76 years N/A	5.5 ± 4.4 years 7.7 ± 4.6 years	ACTRN 12614000287639	Music-based mindfulness interventions techniques (2-h sessions, once a week, for 8 weeks)	No therapy was provided, periodic telephone check-ins (only telephone follow-ups)	PDQ39
Pohl et al. (2020)	RCT	Sweden	Music	EG:23 CG:15	65.38% 70%	69.7 ± 7.0 years 70.4 ± 6.0 years	6.0 ± 4.4 years 6.8 ± 3.6 years	NCT02999997	The Ronnie Gardiner Method, which combines multitasking exercises with visual symbols, coordinated body movements, and verbal synchronization to music rhythm (Two 60-min sessions per week for 12 weeks)	Usual care with no additional activities (No additional intervention)	PDQ39, FES, MoCA
Paolo et al. (2019)	RCT	Italy	Dance	EG:10 CG:9	60% 70%	67.8 ± 5.9 years 67.1 ± 6.3 years	4.4 ± 4.5 years 5 ± 2.9 years	NP/3339	Traditional Sardinian folk dance (Two 90-min sessions per week for 12 weeks)	No additional intervention, continuing with regular medical care (No additional intervention)	MoCA, BDI
Ni et al. (2016)	RCT	USA	MBIs (Yoga)	EG:13 CG:10	73.33% 50%	71.2 ± 6.5 years 74.9 ± 8.3 years	6.9 ± 6.3 years 5.9 ± 6.2 years	N/A	Power yoga (1-h sessions twice a week for 12 weeks)	Participated in health education sessions but no exercise intervention (Monthly health education sessions over 12 weeks)	PDQ39
Modugno et al. (2010)	RCT	Italy	Theater	EG:10 CG:10	60% 50%	63 ± 1.13 years 62 ± 1.58 years	9.4 ± 4.5 years 10 ± 5 years	N/A	The theater workshops (One or two workshops per month, 6 h each, over a period of 3 years)	Physical therapy included muscle stiffness prevention, posture correction, balance training, and gait training (Three weekly sessions, 2–3 h each, over 3 years)	PDQ39
Michels et al. (2018)	RCT	USA	Dance	EG:9 CG:4	66.67% 25%	66.44 years 75.5 years	2.11 ± 0.33 years 2.50 ± 1.00 years	N/A	Dance therapy (60-min sessions once a week for 10 weeks)	The support group involved education and emotional support discussions without any physical activity (Weekly discussions and education sessions without physical activity)	PDQ39, MoCA, BDI
Lee et al. (2018)	RCT	Korea	Dance Qigong	EG:25 CG:16	40% 43.75%	65.8 ± 7.2 years 65.7 ± 6.4 years	4.5 ± 3.3 years 4.4 ± 3.0 years	IRB No. 200902	Turo PD, a hybrid qigong-dance program (60 min per session, twice a week, for 8 weeks)	No intervention during the 8-week period, followed by participation in the same Turo PD program (N/A during the first 8 weeks, then the same program as the Turo PD group)	BDI
Kwok et al. (2019)	RCT	Hong Kong, China	MBIs (Yoga and Mindful-ness)	EG:57 CG:55	52.1% 41.8%	63.7 ± 8.2 years 63.5 ± 9.3 years	3.65 ± 3.37 years	CUHK_ CCRB00522	Mindfulness yoga (90-min sessions, once a week, for 8 weeks)	SRTE, consisting of 60-min group sessions focusing on stretching, resistance training, and warm-up exercises (60-min sessions, once a week, for 8 weeks)	PDQ39, PDQ8, HADS-Anxiety, HADS-Depression
Kwok et al. (2022)	RCT	Hong Kong, China	MBIs (Yoga and mindful-ness)	EG:71 CG:67	52.1% 41.8%	63.7 ± 8.2 years 63.6 ± 9.3 years	N/A	ChiCTR-IOR-16009065	Mindfulness yoga (90-min sessions, once a week for 8 weeks)	Stretching and resistance training exercises, including warm-up exercises, resistance training, and stretching (60-min sessions, once a week for 8 weeks)	PDQ39, PDQ8, HADS-Anxiety, HADS-Depression
Kunkel et al. (2017)	RCT	United Kingdom	Dance	EG:31 CG:15	52.78% 40%	71.3 ± 7.7 years 69.7 ± 6.0 years	4.7 ± 3.5 years 7.0 ± 4.9 years	ISRCTN 63088686	ballroom dancing sessions (Twice a week for 10 weeks)	Control group participants received usual care and were offered dance class vouchers at the end of the study (No intervention during the trial period, usual care during the trial period, usual care)	PDQ39
Haas et al. (2024)	RCT	Brazil	Dance	EG:31 CG:21 CG2:31	EG:67.74% CG:19.05% CG2:25.81%	71.6 ± 8.9 years 66.8 ± 9.0 years CG2:67.9 ± 11.2 years	5.6 ± 5.1 years 8.0 ± 4.7 years CG2:7.0 ± 5.1 years	NCT03370315	Brazilian dance (60-min sessions, twice a week for 12 weeks)	CG: Deep-water running and muscle strength exercises CG2: Nordic walking focusing on coordination and balance with walking poles (60-min sessions, twice a week for 12 weeks)	PDQ39, FES, MoCA
Frisaldi et al. (2021)	RCT	Italy	Dance	EG:19 CG:19	52.63% 68.42%	60.68 ± 6.34 years 61.21 ± 7.18 years	5.99 ± 2.18 years 6.43 ± 2.50 years	N/A	1 h of conventional physiotherapy followed by 1 h of dance class (focused on contemporary dance and ballet without music)(1 h of physiotherapy and 1 h of dance, 3 times a week, for 5 weeks)	physiotherapy followed by another hour of conventional physiotherapy (1 h of physiotherapy, 3 times a week, for 5 weeks)	PDQ39, FES, MoCA, BDI
Cheung et al. (2018)	RCT	USA	MBIs (Yoga)	EG:10 CG:10	50% 50%	63.5 ± 8.5 years 65.8 ± 6.6 years	4.8 ± 2.9 years 4.8 ± 2.9 years	NCT02509610031	Hatha yoga sessions. (60-min sessions, twice weekly, for 12 weeks)	Wait-list control (no intervention during the first 12 weeks) (After 12 weeks, the control group received the same yoga intervention as the experimental group)	PDQ39, MoCA, BDI
Calabrò et al. (2019)	RCT	Italy	Music	EG:25 CG:25	45% 30%	70 ± 8 years 73 ± 8 years	10 ± 3 years 9.3 ± 3 years	NCT03434496	Treadmill gait training without Rhythmic Auditory Stimulation (non-RAS)(30 min per day, 5 times per week, for 8 weeks)	Treadmill gait training without Rhythmic Auditory Stimulation (non-RAS)(30 min per day, 5 times per week, for 8 weeks.)	FES
Buchwitz et al. (2021)	RCT	Germany	MBIs (Mindful-ness)	EG:13 CG:14	57.14% 43.75%	60.5 ± 8.3 years 66.5 ± 6.5 years	N/A	DRKS00015807	A newly developed MBIs (Mindfulness) training program (Eight weekly sessions, approximately 2 h each, for 8 weeks)	Waitlist control with no intervention during the trial period (No intervention during the trial; control group offered mindfulness training after study conclusion)	PDQ39, BDI
Doshi et al. (2025)	RCT	India	Music and dance	EG:15 CG:13	53.3% 69.2%	63.3 ± 7.9 years 66.2 ± 8.3 years	N/A		Dance/music + meditation (60-min sessions, ≥3 times/week for 6 months)	Usual care with routine activities and medication only	PDQ39, BDI
Advocat et al. (2016)	RCT	Australia	MBIs (Mindful-ness)	EG:24 CG:25	66.67% 51.52%	62.8 ± 7.6 years 63.7 ± 8.6 years	N/A	ACTRN 12612000440820	mindfulness-based lifestyle program (2-h sessions, once per week, for 6 weeks)	No intervention during the first 6 weeks (waitlist control) (After 7 weeks, participants received the same 6-week mindfulness-based program)	PDQ39
Lee et al. (2015)	RCT	Republic of Korea	Dance	EG:10 CG:10	50% 50%	EG: 68.4 ± 2.9 CG: 70.1 ± 3.3	N/A	N/A	Neurodevelopment treatment (30 min) + Functional electrical stimulation (15 min) + Virtual reality dance exercise (30 min); 5 times/week; 6 weeks	Neurodevelopment treatment (30 min) + Functional electrical stimulation (15 min); 5 times/week; 6 weeks	BDI

### 3.4 Primary outcomes (art-based interventions)

#### 3.4.1 Fear of falling (FES)

##### 3.4.1.1 Overall analysis

**Figure 3:**
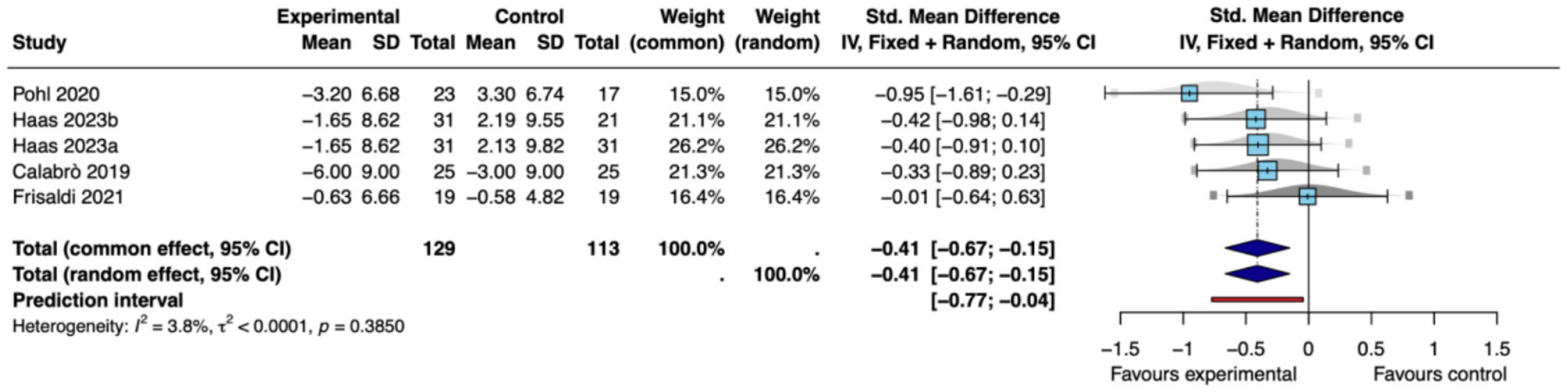
Forest plot of the FES.

The FES measures fear of falling and confidence in performing daily activities, with lower scores indicating greater confidence and reduced fear. Five studies (*n* = 242) reported FES outcomes. Pooled results demonstrated a moderate effect size favoring Art-Based Interventions (*SMD* = −0.41, 95% CI [−0.67, −0.15], *p* < 0.05, PI [−0.78, −0.04]), with low heterogeneity (*I*^2^ = 3.8%) and no evidence of publication bias (Figure 3).

##### 3.4.1.2 Subgroup analysis by intervention type

**Figure 4:**
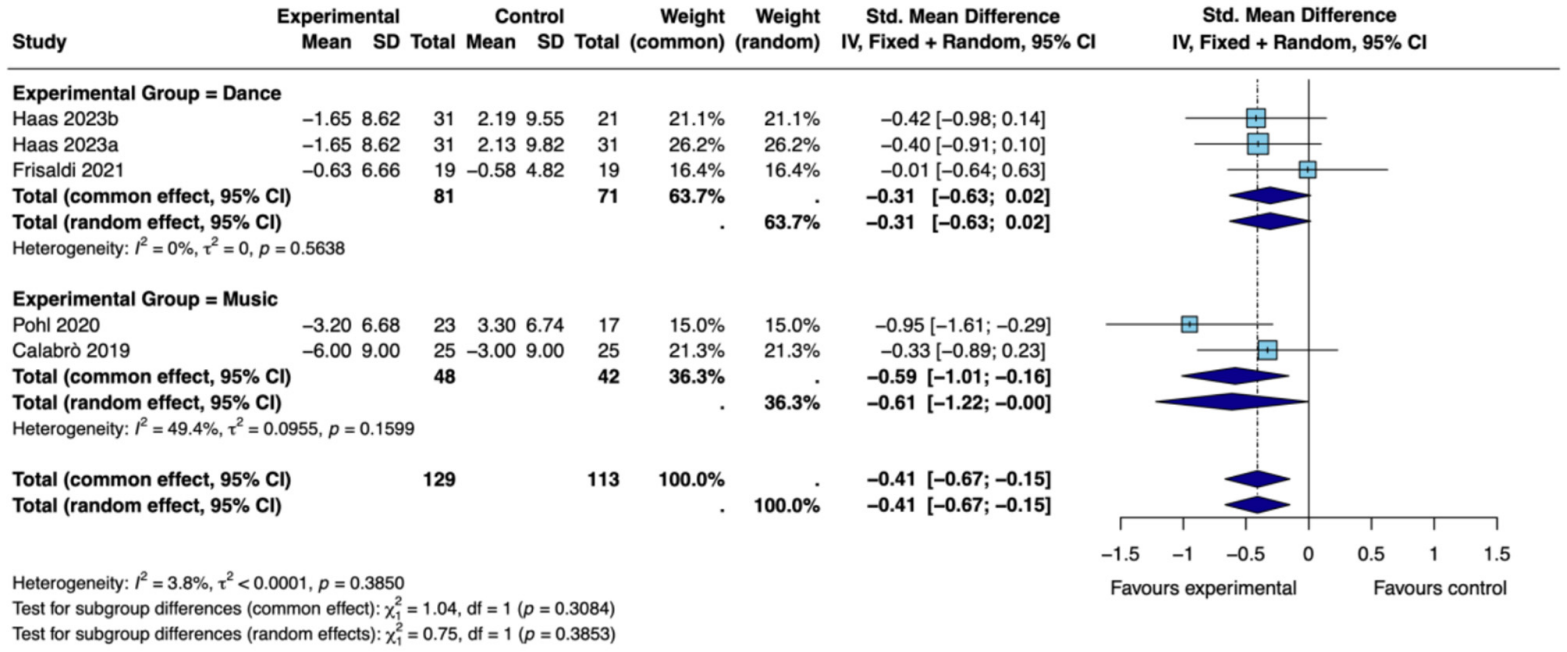
Subgroup analysis of FES outcomes stratified by art-based intervention types.

Subgroup analyses of FES outcomes indicated that dance interventions (three studies) did not yield a statistically significant effect (*SMD* = −0.31, 95% CI [−0.63, 0.02], *p* > 0.05; *I^2^* = 0%). Music interventions (two studies) showed a moderate and statistically significant effect (SMD = −0.61, 95% CI [−1.22, −0.00], p < 0.05; I^2^ = 49.4%), though based on limited evidence. The overall pooled effect (*SMD* = −0.41, 95% CI [−0.67, −0.15], *p* < 0.05; *I^2^* = 3.8%) indicated a statistically significant association with reduced fear of falling among individuals with PD. Subgroup findings should be interpreted cautiously due to limited study numbers (Figure 4).

#### 3.4.2 Quality of life (PDQ-39)

##### 3.4.2.1 Overall analysis

**Figure 5:**
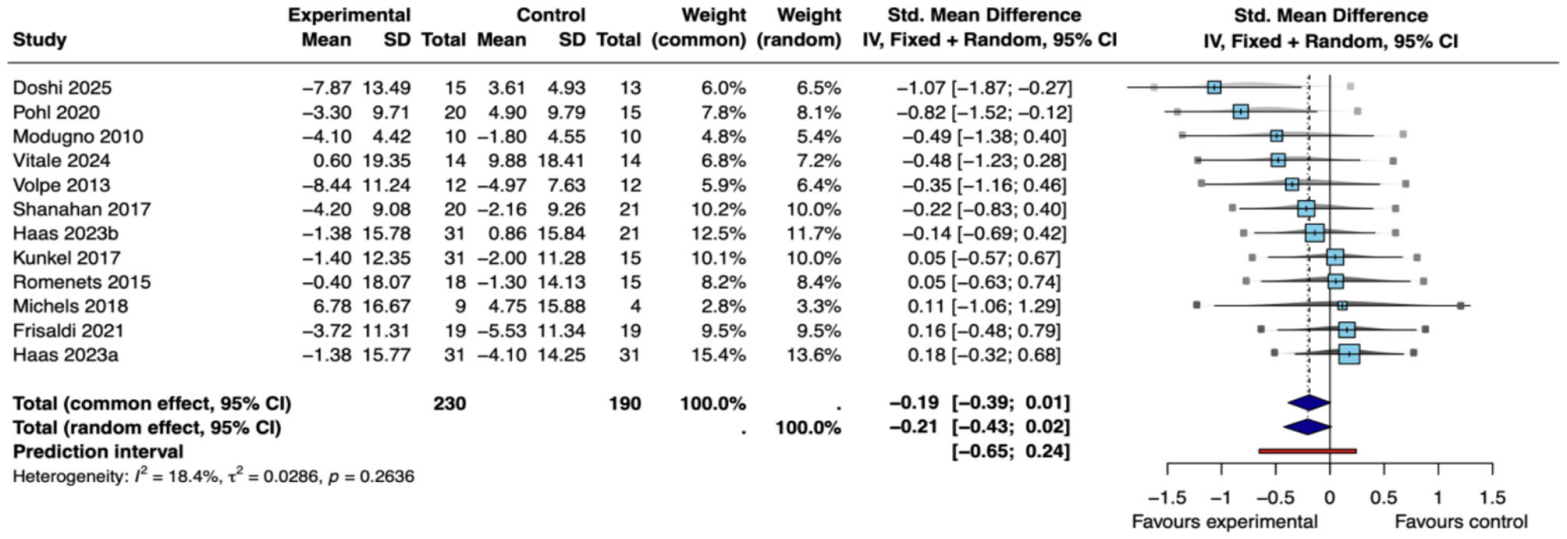
Forest plot of the PDQ-39.

A lower PDQ-39 score indicates better quality of life. 12 studies (*n* = 420) reported PDQ-39 outcomes. The pooled results demonstrated a small, non-significant effect of Art-Based Interventions on quality of life compared with controls (*SMD* = −0.19, 95% CI [−0.39, 0.01], *p* > 0.05), with low heterogeneity (*I*^*2*^ = 18.4%). A leave-one-out sensitivity analysis showed that exclusion of the study by Michels et al. (2018) further reduced between-study heterogeneity, while the overall effect size remained stable. No publication bias was detected according to Egger's test (*p* = 0.212) (Figure 5).

##### 3.4.2.2 Subgroup analysis by intervention type

Dance interventions showed a small, non-significant effect on quality of life (*SMD* = −0.05, 95% CI [−0.26, 0.17], *p* = 0.579), while the Theater intervention showed a moderate but non-significant effect (*SMD* = −0.49, 95% CI [−1.38, 0.40], *p* = 0.281).

**Figure 6:**
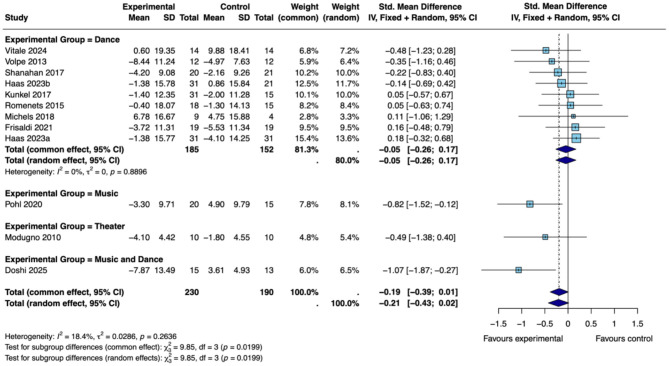
Subgroup analysis of PDQ-39 outcomes stratified by art-based intervention types.

Single Music and Music-and-Dance interventions yielded the largest point estimates (Music: *SMD* = −0.82, 95% CI [−1.52, −0.12]; Music-and-Dance: SMD = −1.07, 95% CI [−1.87, −0.27]). However, as both estimates were derived from single randomized controlled trials, they should be interpreted with caution. Overall, pooled effects for dance and theater interventions were not statistically significant, whereas interventions incorporating music… showed comparatively larger point estimates, although these findings were derived from single trials and remain exploratory for individuals with Parkinson's disease (Figure 6).

#### 3.4.3 Depression (BDI)

Eight studies (*n* = 220) evaluated depressive symptoms using the Beck Depression Inventory (BDI). The pooled results demonstrated a small but non-significant effect of art-based interventions on depressive symptoms (*SMD* = −0.23, 95% CI [−0.63, 0.18], *p* > 0.05).

**Figure 7:**
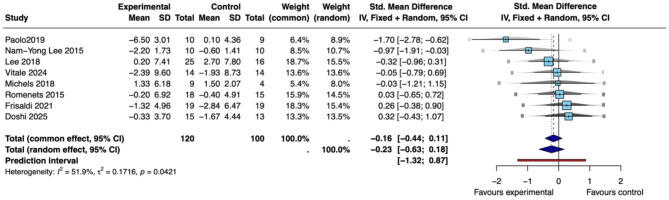
Forest plot of the BDI.

**Figure 8:**
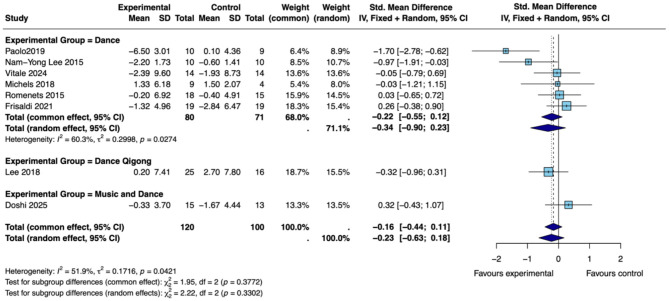
Subgroup analysis of BDI outcomes stratified by art-based intervention types.

Subgroup analyses likewise showed no statistically significant differences across intervention types, including Dance interventions (*SMD* = −0.34, 95% CI [−0.90, 0.23], *p* > 0.05), Dance and Qigong interventions (*SMD* = −0.32, 95% CI [−0.96, 0.31], *p* > 0.05), and Music-and-Dance interventions (*SMD* = 0.32, 95% CI [−0.43, 1.07], *p* > 0.05) (Figures 7, 8).

#### 3.4.4 Cognition (MoCA)

**Figure 9:**
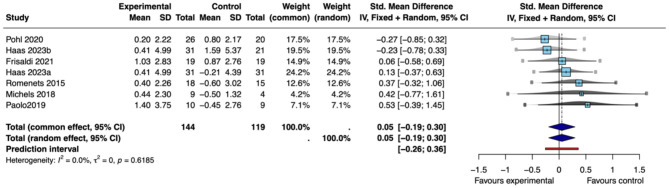
Forest plot of the MoCA.

**Figure 10:**
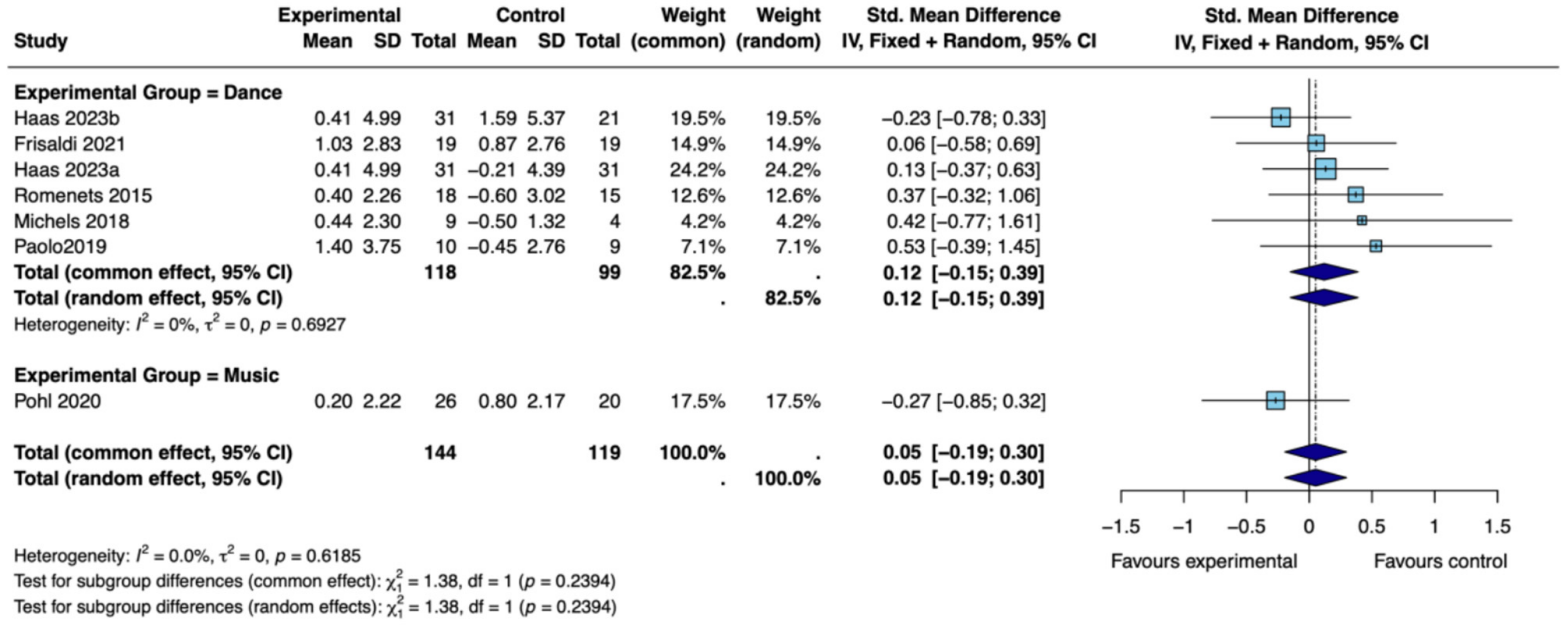
Subgroup analysis of MoCA outcomes stratified by art-based intervention types.

Seven studies (*n* = 263) assessed cognitive function using the MoCA. The pooled analysis indicated no statistically significant effect of Art-Based Interventions on cognition (*SMD* = 0.05, 95% CI [−0.19; 0.30], *p* > 0.05), with subgroup analyses(Dance: *SMD* = 0.12, 95% CI[−0.15; 0.39], *p* > 0.05; Music: *SMD* = −0.27, 95% CI [−0.85; 0.32], *p* > 0.05) across intervention types also showing no significant results (Figures 9, 10).

### 3.5 Secondary outcomes (yoga and mindfulness)

Although yoga and mindfulness are generally classified as mind body interventions rather than art interventions, we identified that yoga- and mindfulness-based components were incorporated in some studies within broader arts-based or embodied practice contexts (e.g., the Ponzio Arts interventions program at Children's Hospital Colorado, which integrates yoga into arts interventions). These interventions share certain features with art-based approaches, which are relevant to non-motor symptom management in Parkinson's disease. Given this conceptual overlap, yoga and mindfulness interventions were included as secondary outcomes to provide complementary evidence. To avoid confounding the primary effects of art-based interventions, these studies were not pooled with the main analyses, but were instead analyzed and reported separately. This approach allows a clearer interpretation of the primary findings while offering additional context regarding related mind-body interventions.

#### 3.5.1 Parkinson's disease questionnaire-39 (PDQ-39)

**Figure 11:**
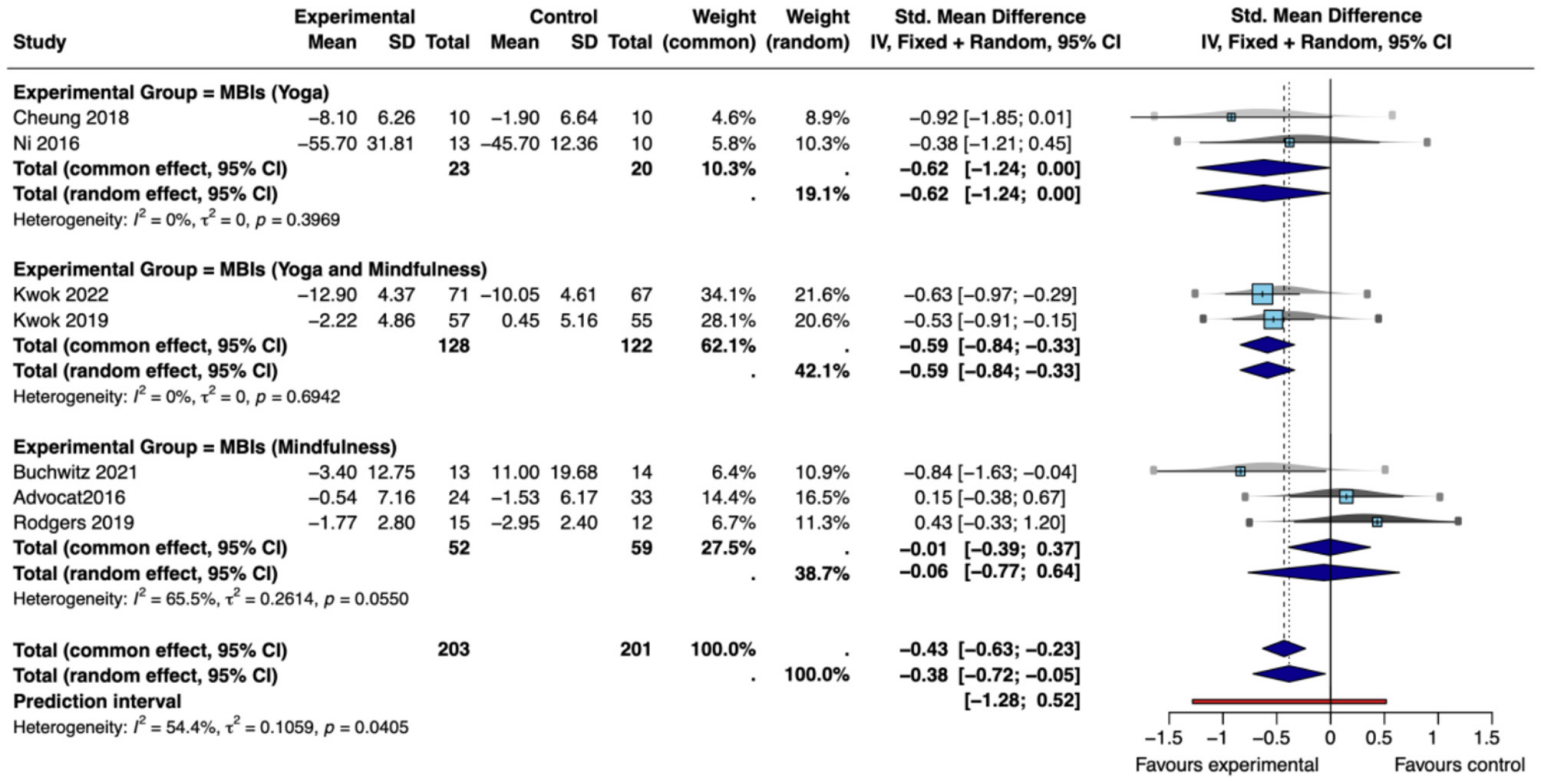
Forest plot of the PDQ-39 outcomes stratified by yoga and mindfulness intervention types.

Health-related quality of life was primarily assessed using the PDQ-39. Seven studies (*n* = 404) reported PDQ-39 outcomes. Pooled analysis suggested a small-to-moderate effect reaching statistical significance following yoga and mindfulness interventions (SMD = −0.38, 95% CI [−0.72, −0.05], *p* = 0.026), with moderate heterogeneity (I^2^ = 54.4%). A leave-one-out sensitivity analysis showed that excluding Rodgers et al. (2019) reduced heterogeneity from 54.4% to 33.4%, while the pooled effect estimate remained stable (Figure 11).

#### 3.5.2 Parkinson's disease questionnaire-8 (PDQ-8)

**Figure 12:**
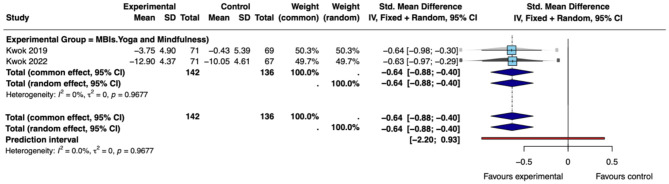
Forest plot of the PDQ-8 outcomes stratified by yoga and mindfulness intervention types.

Two studies (*n* = 278) assessed quality of life using the PDQ-8. The pooled results suggested a moderate effect estimate reaching statistical significance in favor of yoga and mindfulness interventions (SMD = −0.64, 95% CI [−0.88, −0.40], *p* < 0.01), with no observed heterogeneity (I^2^ = 0%). The prediction interval did not cross the null, given that this finding is based on only two studies, it should be interpreted cautiously. The PDQ-8 is a short-form version of the PDQ-39 that captures core domains of health-related quality of life and was therefore analyzed alongside PDQ-39 to allow complementary assessment across studies using different questionnaire formats (Figure 12).

#### 3.5.3 Anxiety and depression (HADS)

**Figure 13:**
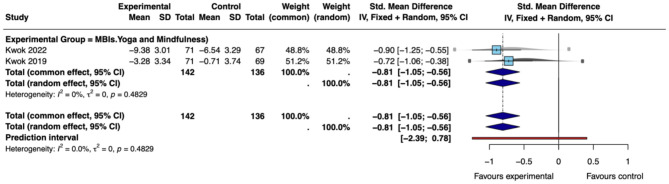
Forest plot of the HADS-Anxiety outcomes stratified by yoga and mindfulness intervention types.

**Figure 14:**
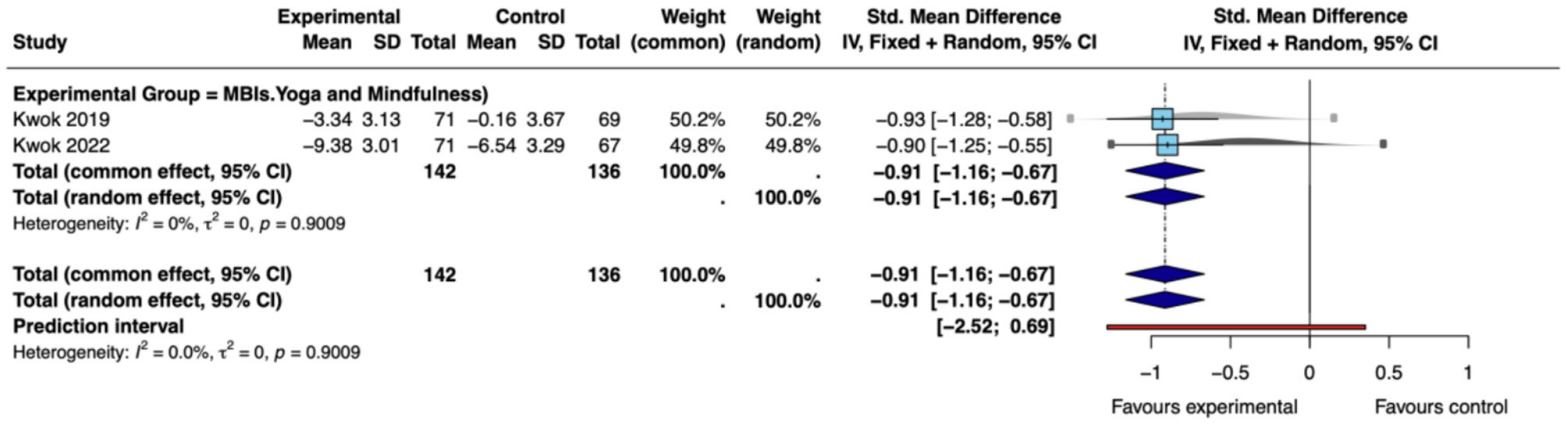
Forest plot of the HADS-Depression outcomes stratified by yoga and mindfulness intervention types.

Anxiety and depressive symptoms were evaluated using the Hospital Anxiety and Depression Scale (HADS). Only two studies (*n* = 278) contributed data for each outcome. Yoga and mindfulness interventions were associated with large effect estimates reaching statistical significance for both anxiety (SMD = −0.81, 95% CI [−1.05, −0.56], *p* < 0.01) and depression (SMD = −0.91, 95% CI [−1.16, −0.67], p < 0.01), with no heterogeneity detected (I^2^ = 0% for both outcomes); however, these results are derived from a very limited evidence base and should be interpreted with caution (Figures 13, 14).

### 3.6 Prediction interval

Prediction intervals were calculated under a random-effects model using the τ^2^ estimate derived from the REML method to assess the expected range of effects in future comparable studies. Among all outcomes, both the Falls Efficacy Scale (FES) and PDQ-8 exhibited 95% prediction intervals that did not cross the null value, indicating relatively consistent and potentially generalizable benefits of art-based interventions in reducing fear of falling and improving health-related quality of life, respectively. prediction intervals for the remaining outcomes—including PDQ-39, HADS anxiety, HADS depression, BDI, and MoCA—crossed zero, suggesting substantial between-study heterogeneity and context-dependent effects. These findings imply that, although pooled effects may reach statistical significance for some outcomes, the magnitude and direction of effects may vary across future settings. Overall, the presence of non-null prediction intervals for FES and PDQ-8 provides stronger evidence for the robustness of art-based interventions on confidence-related and short-form quality-of-life outcomes. However, the limited number of contributing studies underscores the need for larger, well-designed, and methodologically standardized randomized controlled trials to confirm the stability and reproducibility of these effects (Table 4).

**Table 4:** Pooled effect sizes for non-motor outcomes.

**Intervention type**	***k***	***n***	**SMD (95% CI)**	**I^2^ (%)**	***p*-value**	**Prediction interval**
**Art-based interventions**						
FES	5	242	−0.41 (−0.67, −0.15)	3.8	0.002	Excludes 0
Dance	3	152	−0.31 (−0.63, 0.02)	0	0.061	/
Music	2	90	−0.61 (−1.22, −0.01)	49.4	0.048	/
PDQ-39	12	420	−0.19 (−0.39, 0.01)	18.4	0.058	Includes 0
Dance	9	337	−0.05 (−0.26, 0.17)	0	0.579	/
Music	1	35	−0.82 (−1.52, −0.12)	0	0.022	/
Theater	1	20	−0.49 (−1.38, 0.40)	0	0.281	/
Music + Dance	1	28	−1.07 (−1.87, −0.27)	0	0.009	/
BDI	8	220	−0.23 (−0.63, 0.18)	51.9	0.239	Includes 0
MoCA	7	263	0.05 (−0.19, 0.30)	0	0.683	Includes 0
**Yoga and mindfulness**						
PDQ-8	2	278	−0.64 (−0.88, −0.40)	0	0.001	Excludes 0
HADS Anxiety	2	278	−0.81 (−1.05, −0.56)	0	0.005	Includes 0
HADS Depression	2	278	−0.91 (−1.16, −0.67)	0	0.005	Includes 0
PDQ-39	7	404	−0.38 (−0.72, −0.05)	54	0.026	Includes 0

## 4 Discussion

This systematic review and meta-analysis indicate that art-based interventions are associated with selective improvements in non-motor outcomes in PD. Among all outcomes examined, FES demonstrated the most consistent and statistically robust improvement, supported by low heterogeneity, sensitivity analyses, and a prediction interval that excluded the null, suggesting a comparatively stable effect within the current evidence base. pooled effects on quality of life, cognition, and depressive symptoms were small or non-significant, with subgroup findings largely exploratory.

The prominence of fear of falling (FOF) in the present findings is clinically relevant in the context of PD. Falls are highly prevalent in this population, with approximately 60% of individuals experiencing at least one fall annually and nearly two-thirds of these reporting recurrent falls (Latt et al., 2009; Allen et al., 2013; Bloem et al., 2001). This rate is roughly double that observed in the general older population (Bloem et al., 2001). Beyond physical injury and pain, falls contribute to activity restriction and elevated fear of falling, which negatively affect overall health and well-being (Wielinski et al., 2005; Temlett and Thompson, 2006).

FOF has been identified as a major concern in PD, with a subset of individuals describing it as their most distressing physical symptom (Frazier, 2000). Previous studies indicate that FOF predicts future falls and near-falls even in early disease stages (Lindholm et al., 2015) and is associated with reduced community ambulation (Elbers et al., 2013), avoidance of exercise (Ellis et al., 2013), and restricted participation in meaningful activities (Thordardottir et al., 2014). FOF is also consistently linked to activity limitations and physical inactivity (Kader et al., 2016; Bryant et al., 2015). These established relationships suggest that reductions in FOF may be related to improvements in fear-related self-efficacy and behavioral avoidance, domains that strongly influence functional engagement in PD yet are not primary targets of pharmacological management. In this context, improvements in FOF may be clinically meaningful, as lower fear levels have been associated with greater activity participation and reduced movement avoidance. By contrast, effects on cognition, depressive symptoms, and overall quality of life were less consistent. These outcomes were frequently secondary endpoints, and many included trials were not specifically designed or powered to target cognitive or affective change. Additionally, several included music-based trials incorporated rhythmic cueing and structured group engagement, which may relate to the observed subgroup signal, although these design elements were not consistently reported. intervention duration was often short, and heterogeneity in modality, intensity, and delivery format may have diluted pooled effects. Subgroup signals—particularly for music-based interventions—were derived from a limited number of studies and should therefore be considered exploratory.

Several methodological factors warrant careful consideration. Blinding of participants and interventionists is typically infeasible in art-based randomized controlled trials, reflecting a common challenge in behavioral intervention research. This structural limitation may increase susceptibility to expectancy and contextual effects, especially for subjective outcomes such as FOF and quality of life. Accordingly, findings based on patient-reported measures should be interpreted cautiously. To address potential bias, multiple complementary strategies were applied, including leave-one-out sensitivity analyses, publication bias assessment where feasible, prediction interval estimation, and subgroup exploration. Yoga- and mindfulness-based interventions were analyzed separately to avoid conceptual overlap with primary art-based interventions. Together, these approaches support the internal consistency of the main findings while underscoring the need for cautious interpretation.

Although prediction intervals were incorporated to enhance clinical interpretability, most outcomes showed intervals crossing the null, indicating between-study uncertainty. Thus, conclusions regarding generalizability should primarily rely on the comparatively robust finding for fear of falling rather than broader non-motor domains. Although PDQ-8 showed a non-null prediction interval, these results were derived from secondary analyses of yoga and mindfulness interventions, which were not part of the primary art-based intervention framework of this review. Therefore, conclusions regarding robustness and generalizability were based primarily on outcomes derived from the core art-based intervention analyses.

## 5 Future research direction

Although the current evidence base is dominated by dance and music interventions, other art-based modalities—such as drama-based approaches and visual arts including painting—remain markedly underrepresented. The limited number of studies examining these modalities often feature small samples, heterogeneous protocols, or inadequate control conditions, precluding quantitative synthesis.

This scarcity should not be interpreted as evidence of limited therapeutic value, but rather reflects the methodological challenges inherent in evaluating experiential and expressive interventions within conventional randomized controlled trial frameworks. Future research should adopt rigorous yet flexible designs, such as mixed-methods approaches, mechanistic assessments, or adaptive trial designs, to better capture the therapeutic processes and potential benefits of underexplored art-based interventions while maintaining methodological rigor.

## 6 Limitation

Several Despite these findings, several methodological and evidence-related limitations should be acknowledged when interpreting the results. First, outcome measures were not prespecified a priori. All eligible non-motor outcomes were extracted, and only those reported in at least three independent trials were synthesized to ensure statistical stability, which may have limited the range of outcomes included. Second, the evidence base consists of previously published randomized controlled trials with substantial variation in methodological rigor, participant characteristics, intervention delivery, and outcome reporting, thereby constraining comparability across studies (de Natale et al., 2017; Bega et al., 2017). The number of trials specifically targeting non-motor symptoms in Parkinson's disease remains limited, and restriction to English-language publications may have introduced language bias, although it ensured consistency in quality appraisal. Third, art-based interventions varied widely in modality, structure, frequency, and intensity, making it difficult to identify which intervention components were primarily responsible for observed effects. Heterogeneity may also reflect conceptual and methodological differences across intervention types, intervention intensity, and control conditions, which may limit the interpretability and generalizability of the pooled estimates. In addition, the predominance of self-reported outcomes and the inherent infeasibility of double-blinding in art-based randomized controlled trials, a common challenge in behavioral intervention research, may increase susceptibility to reporting and performance bias (Boutron et al., 2017). Finally, most included studies lacked long-term follow-up, precluding conclusions regarding the durability of intervention effects (Rios Romenets et al., 2015; Rodgers et al., 2019). Future studies employing more standardized protocols, blinded outcome assessment where feasible, and longitudinal designs are needed to strengthen the evidence base.

## 7 Conclusion

This systematic review and meta-analysis indicate that art-based interventions show selective effects on non-motor outcomes in Parkinson's disease. FES demonstrated the most consistent and statistically robust improvement, supported by low heterogeneity, sensitivity analyses, and a prediction interval excluding the null. pooled effects on quality of life, cognitive function, and depressive symptoms were small or non-significant, with subgroup findings largely exploratory. Secondary analyses of yoga and mindfulness interventions showed improvements in quality-of-life and affective outcomes but were analyzed separately and not pooled with art-based interventions. Overall, current evidence suggests that art-based interventions may offer adjunctive benefit primarily in fear-related self-efficacy, while effects on broader non-motor domains remain inconclusive. Further well-designed and adequately powered trials are needed to confirm clinical relevance.

Importantly, fear of falling represents a cross-cutting behavioral and psychological construct across stages of Parkinson's disease. Although generalization beyond ambulatory populations should be made with caution, these findings may offer limited, context-specific reference for understanding fear-related self-efficacy and activity engagement within the broader non-motor symptom framework.

## Data Availability

The original contributions presented in the study are included in the article/supplementary material, further inquiries can be directed to the corresponding author.
